# Supplementary material for: Characterization and Diversity of Klebsiella pneumoniae Prophages
Source: Int J Mol Sci. 2023 May 23;24(11):9116. doi: 10.3390/ijms24119116 (PMC10252525; doi:10.3390/ijms24119116)
Supplement: Supplementary file 1 [file ijms-24-09116-s001.zip › ijms-2320228-supplementary.pdf]

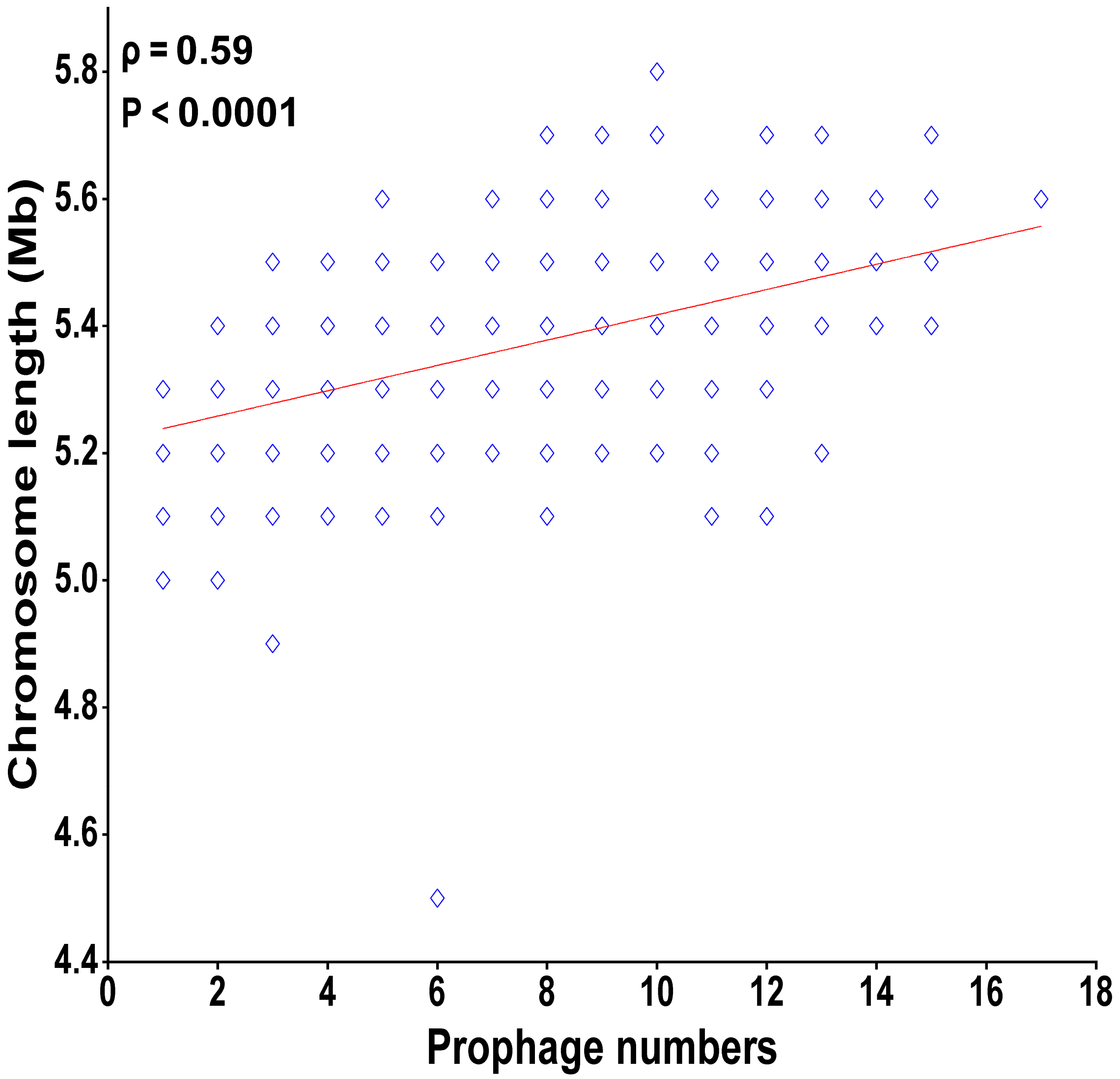

Figure S1: Correlation between the number of prophages per chromosome and the chromosome length of the strains.

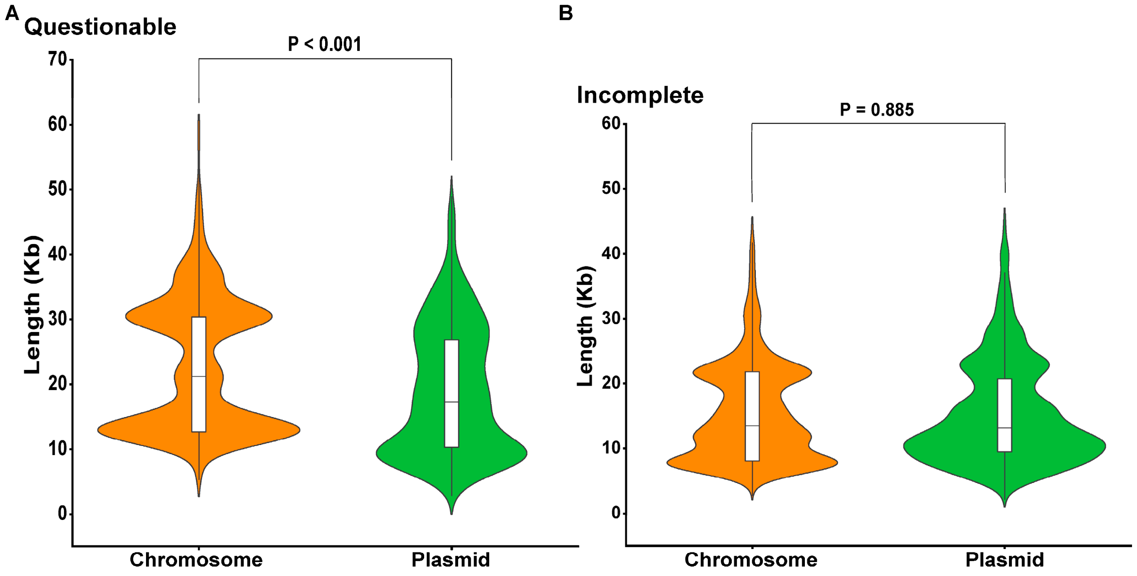

Figure S2: Comparison of the lengths of questionable (A) and incomplete (B) prophages on different localizations of *K. pneumoniae*.

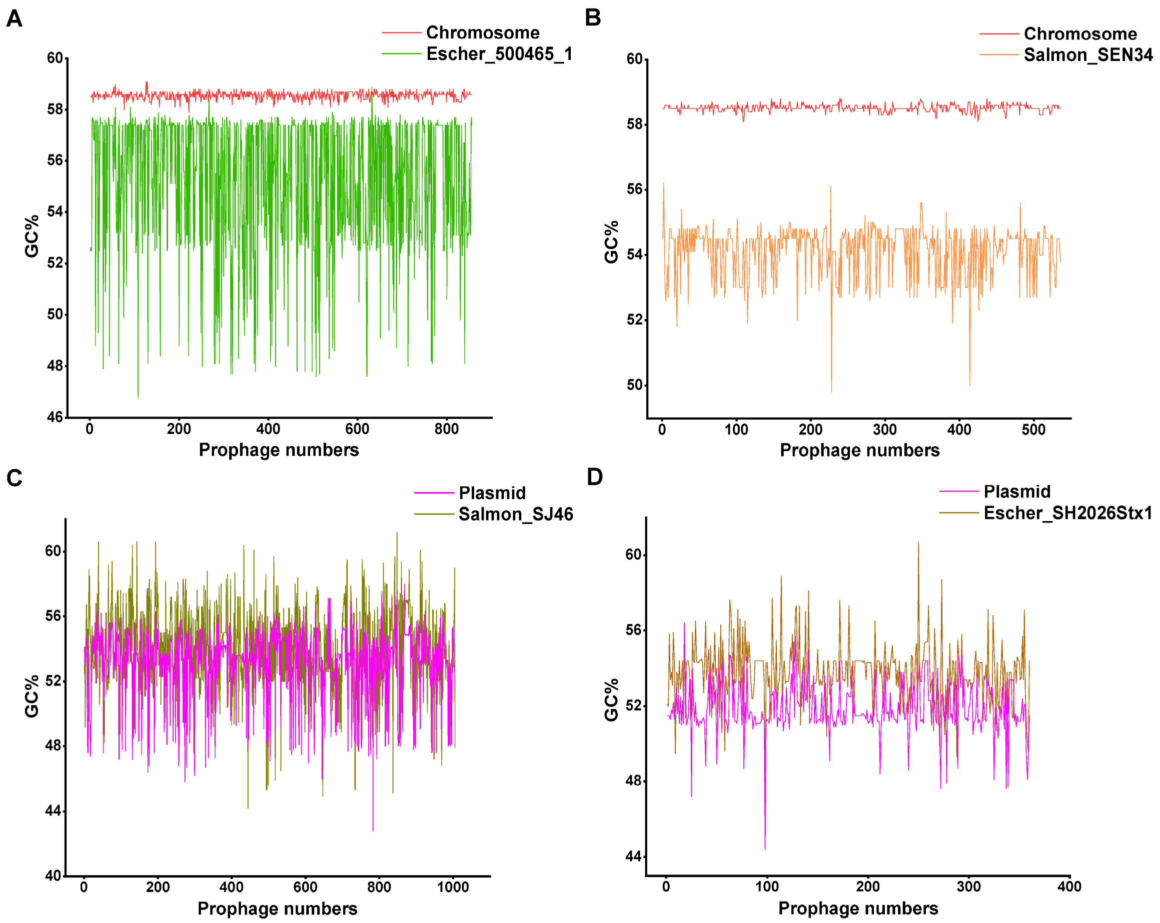

Figure S3: (A-D) Comparison of the GC content of the prophage and the chromosome or plasmid on which it was located.

Table S1: All prophages used in this study; Table S2: *K. pneumoniae* strains and their typing with prophages and their types; Table S3: GC content of different genetic regions of the *K. pneumoniae* strain genome with the GC content of its integrated prophage; Table S4: Plasmids with only one intact prophage, which accounts for 90-100% of the plasmid length; Table S5: A comparative overview of the length and VF category and resistance mechanisms of prophages in chromosomes and plasmids; Table S6: Comparative overview of the GC content of the prophage and the chromosome or plasmid in which they were located.
