# Supplementary material for: Characterization and Diversity of Klebsiella pneumoniae Prophages
Source: Int J Mol Sci. 2023 May 23;24(11):9116. doi: 10.3390/ijms24119116 (PMC10252525; doi:10.3390/ijms24119116)
Supplement: Supplementary file 1 [file ijms-24-09116-s001.zip › Supplementary Table S5.pdf]

Table S5: A comparative overview of the length and VF category and resistance mechanisms of prophages in chromosomes and plasmids.

Localization of prophages	Average length of chromosomes or plasmids with different numbers of prophages (Mb)			Average length of prophages with different degrees of completeness (Kb)			Proportion of prophages with VF or AMR genes (%)				Unique VF category	Main resistance mechanism
	0	1	>=2	Intact	Questionable	Incomplete	No	VF	AMR	VF+AMR		
Chromosome	-	5.21	5.35	46.60	23.01	15.48	68.91	10.85	11.31	8.93	antimicrobial activity or competitive advantage	antibiotic target alteration and antibiotic efflux
Plasmid	0.01	0.09	0.18	42.97	18.95	15.42	56.23	5.12	36.02	2.63	exoenzyme and invasion	antibiotic inactivation
