# Supplementary material for: Characterization and Diversity of Klebsiella pneumoniae Prophages
Source: Int J Mol Sci. 2023 May 23;24(11):9116. doi: 10.3390/ijms24119116 (PMC10252525; doi:10.3390/ijms24119116)
Supplement: Supplementary file 1 [file ijms-24-09116-s001.zip › Supplementary Table S6.pdf]

Table S6: Comparative overview of the GC content of the prophage and the chromosome or plasmid in which they were located.

Localization of prophages	Average GC content (%)[1]					
	**Entero_P4**	**Escher_500465_1**	**Salmon_SEN34**	**Escher_RCS47**	**Salmon_SJ46**	**Escher_SH2026Stx1**
Chromosome	58.5/53.7	58.6/55.2	58.5/54.1	58.5/52.2	NC	NC
Plasmid	NA	NC	NC	53.1/54.4	53.1/54.0	51.9/53.8

[1] The numbers in the table are the average GC content of chromosomes or plasmids/average GC content of prophages. NA, not available; NC, not compared.
